# Supplementary material for: Unilateral Strength Training Imparts a Cross-Education Effect in Unilateral Knee Osteoarthritis Patients
Source: J Funct Morphol Kinesiol. 2022 Sep 28;7(4):77. doi: 10.3390/jfmk7040077 (PMC9589957; doi:10.3390/jfmk7040077)
Supplement: Supplementary file 1 [file jfmk-07-00077-s001.zip › jfmk-1891350-Supplementary.pdf]

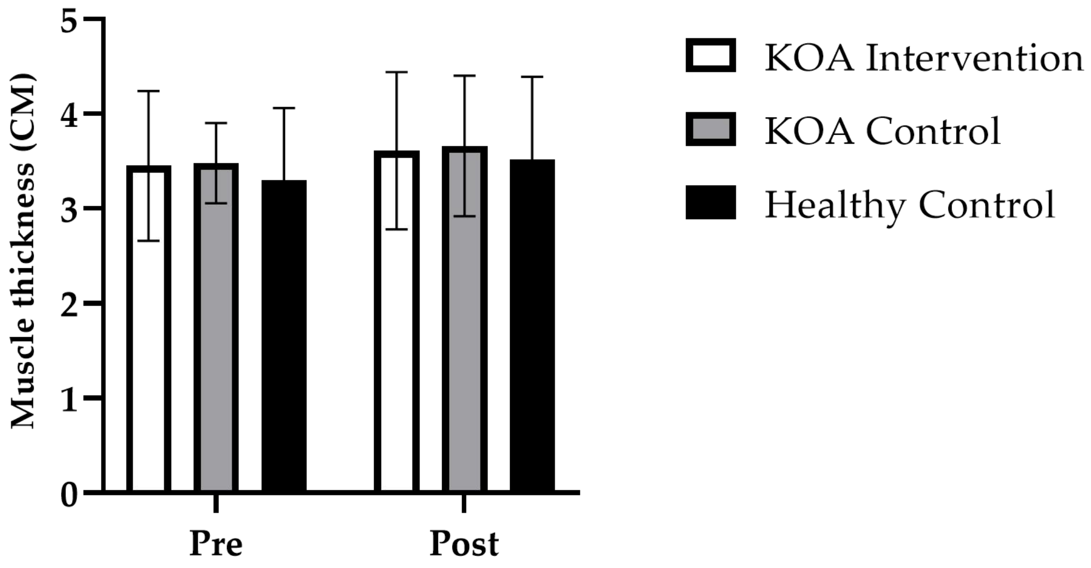

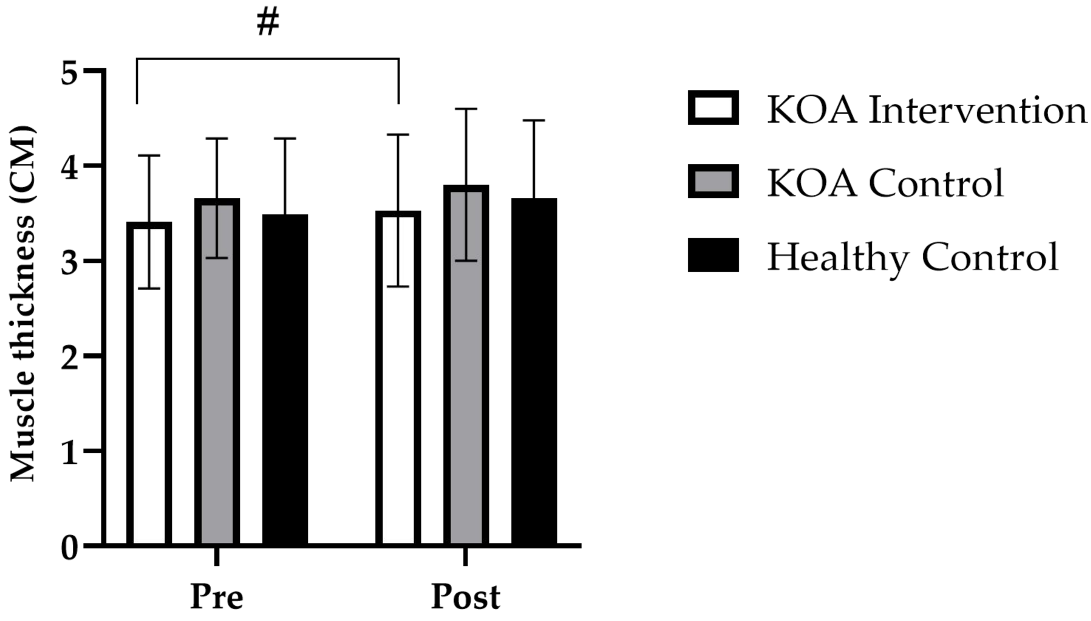

Figure S1. To graph shows muscle thickness of the trained limb, whilst the bottom graph displays muscle thickness of the untrained limb. # Significant difference pre-post in muscle thickness for the untrained limb for the KOA intervention group.
